# Giant bypass aneurysm, a cause of suspected cardiac mass

**DOI:** 10.1186/2193-1801-3-433

**Published:** 2014-08-13

**Authors:** Jan M Sohns, Martin Fasshauer, Wieland Staab, Michael Steinmetz, Joachim Lotz, Christina Unterberg-Buchwald

**Affiliations:** Institute for Diagnostic and Interventional Radiology, Center of Radiology, DZHK, Georg-August-University Göttingen, UMG Universitätsmedizin Göttingen, Robert-Koch-Str. 40, 37075 Göttingen, Germany; German Centre for Cardiovascular Research, DZHK, Göttingen, Germany; Department of Cardiology and Pneumology, Georg-August-University, Göttingen, Germany; Department of Pediatric Cardiology and Intensive Care Medicine, Georg-August-University, Göttingen, Germany

**Keywords:** Cardiac bypass aneurysm, Cardiac MRI, Bypass thrombus, Cardiac mass, Cardiopulmonary bypass, Aneurysm

## Abstract

**Introduction:**

A 66-years old man suffering from coronary artery disease appeared without symptoms for routine follow-up in our clinic.

**Case description:**

The echocardiogram revealed a tumorous mass of the right atrium and right ventricle. In the past, coronary revascularization with venous grafts of the right coronary artery and circumflex artery as well as internal mammaria graft to the left anterior descending artery was performed 20 years before. The general clinicians presented the case to the surgeons and it was decided to perform cardiac MRI as a preoperative diagnostic modality.

**Discussion and evaluation:**

Following cardiac magnetic resonance imaging (MRI) showed a mass in the pericardium in the right atrioventricular groove with thrombotic material. Due to the MRI the patient underwent coronary angiography to confirm an aneurysm.

**Conclusions:**

The learning points from this case are that cardiac MRI is a very useful tool for further evaluation of suspected cardiac masses and should be performed for further characterization.

## Background and case description

**Figure 1:**
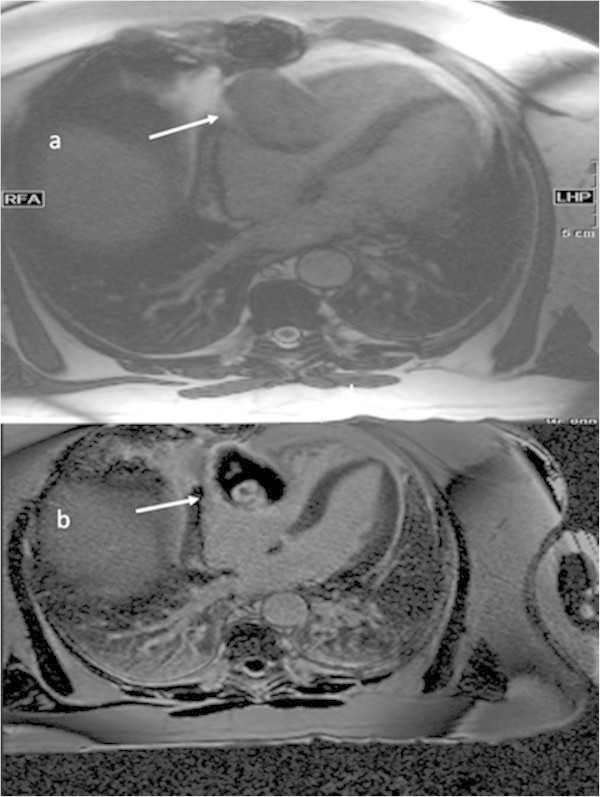
**Aneurysm in MRI.** **a**: True-FISP-sequence demonstrates an extra-pericardial mass, which compresses the atrio-ventricular groove (white arrow, transversal views, TR: 28.8, TE: 1.22). **b**: After intravenous gadolinium application, partial enhancement of the thrombosed aneurysm is seen in detail (TR: 650, TE: 1.2, T1-sequences with contrast medium, transversal view).

**Figure 2:**
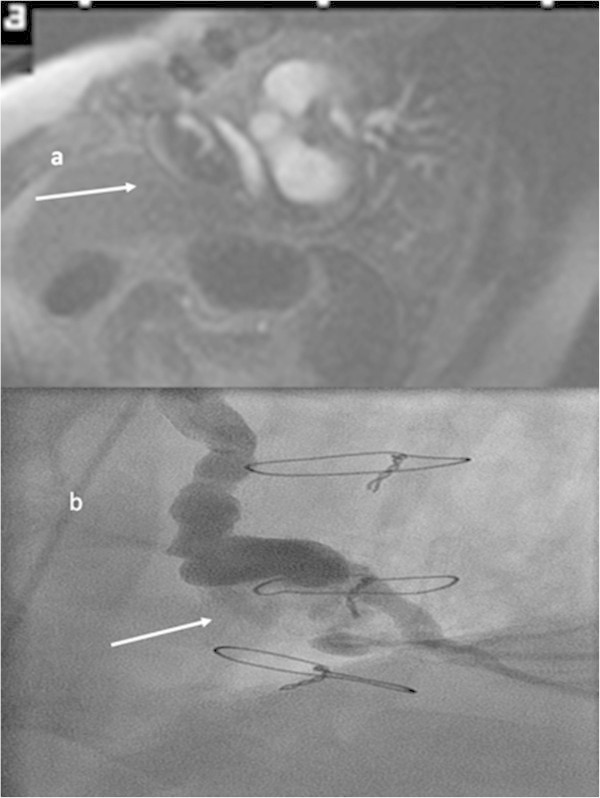
**Aneurysm in MRI and coronary angiography. a**: Perfusion of the aorta and the bypass aneurysm is seen at the same time (white arrows, parasagittal views, application of contrast medium, TR: 174, TE: 0.94). **b**: Coronary angiography (LAO 30 0) revealed a large aneurysm of the degenerated venous graft of the right coronary artery (RCA) with a thrombus and small leakage into the ventricle (white arrow). The distal part of the native RCA (occluded at the ostium) is promptly perfused with the graft.

Spontaneous bypass aneurysm is a rare complication of coronary bypass revascularization (Hiraoka et al. 2012; Ebina et al. 2009; Kalimi et al. 1999; Seto et al. 2008; Mangia et al. 2012). Generally, it occurs as a degenerative process of the graft or after coronary intervention. Clinical presentation could be angina pectoris or myocardial infarction due to distal embolization. In rare cases the aneurysm compresses a heart chamber with the consequence of cardiac decompensation or it could even perforate (Berdajs et al. 2011; Tran et al. 2005; Yohann et al. 2000). In our case the patient did not present any clinical symptoms. The aneurysm was detected during a routine echocardiogram and was supposed to be a cardiac mass. Following cardiac magnetic resonance imaging (MRI) showed a mass in the pericardium in the right atrioventricular groove with thrombotic material (6.5 × 4 cm) (Figure 1). Perfusion images revealed that the suspected mass was enhanced with contrast medium at the same moment like the aorta and it was part of the venous bypass graft of the right coronary artery. Additionally, late gadolinium enhancement showed a transmural baso-inferior scar in the territory of the right coronary artery in the myocardium. Medical history could not reveal whether this scar preexisted before bypass surgery or developed afterwards (18 ml Gadovist, Bayer Healthcare, Leverkusen, Germany). However, the localization was not typical for distal bypass embolization. Due to the MRI the patient underwent coronary angiography to confirm the aneurysm (Figure 2). Furthermore, a small fistula of the aneurysm into the right ventricle was suspected. As the patient was asymptomatic and without any signs of ischemia (normal cycle ergometer), he refused re-operation and is still without ischemia 18 months after imaging. The ejection fraction in cardiac MRI was about 54%, septal thickness 10 mm, end-diastolic volume of the left ventricle 115 ml, end-systolic volume of the left ventricle 53 ml, stroke volume 62 ml, and cardiac output 4.4 l/min.

## Discussion and conclusive evaluation

The learning points from this case are that cardiac MRI is a very useful tool for further evaluation of suspected cardiac masses and should be performed for further characterization and planning of therapeutical options, particularly after previous cardiac interventions (Hiraoka et al. 2012; Ebina et al. 2009; Kalimi et al. 1999; Seto et al. 2008; Mangia et al. 2012; Berdajs et al. 2011; Tran et al. 2005; Yohann et al. 2000).

## Patient’s consent

Written informed consent was obtained from the patient for publication of this case report and any accompanying images. A copy of the written consent is available for review by the Editor of this journal.

## Abbreviations

MRI: Magnetic resonance imaging
RCA: Right coronary artery
TE: Time of echo
TR: Time of repetition
True-FISP: True fast imaging with steady state precession.
